# Aging, Cell Senescence, the Pathogenesis and Targeted Therapies of Osteoarthritis

**DOI:** 10.3389/fphar.2021.728100

**Published:** 2021-08-23

**Authors:** Xin-Xin Zhang, Shi-Hao He, Xu Liang, Wei Li, Tian-Fang Li, Dai-Feng Li

**Affiliations:** ^1^ Department of Rheumatology, The First Affiliated Hospital of Zhengzhou University, Zhengzhou, China; ^2^ Department of Orthopaedics, The First Affiliated Hospital of Zhengzhou University, Zhengzhou, China; ^3^ Department of Magnetic Resonance Imaging, Henan Key Laboratory of Functional Magnetic Resonance Imaging and Molecular Imaging, The First Affiliated Hospital of Zhengzhou University, Zhengzhou, China

**Keywords:** osteoarthritis, aging, senescence, cartilage, cells

## Abstract

Osteoarthritis (OA) is a chronic, debilitating joint disease characterized by progressive destruction of articular cartilage. For a long time, OA has been considered as a degenerative disease, while recent observations indicate the mechanisms responsible for the pathogenesis of OA are multifaceted. Aging is a key factor in its development. Current treatments are palliative and no disease modifying anti-osteoarthritis drugs (DMOADs) are available. In addition to articular cartilage degradation, cellular senescence, synovial inflammation, and epigenetic alterations may all have a role in its formation. Accumulating data demonstrate a clear relationship between the senescence of articular chondrocytes and OA formation and progression. Inhibition of cell senescence may help identify new agents with the properties of DMOADs. Several anti-cellular senescence strategies have been proposed and these include sirtuin-activating compounds (STACs), senolytics, and senomorphics drugs. These agents may selectively remove senescent cells or ameliorate their harmful effects. The results from preclinical experiments and clinical trials are inspiring. However, more studies are warranted to confirm their efficacy, safety profiles and adverse effects of these agents.

## Introduction

Osteoarthritis (OA) has surpassed diabetes and cardiovascular diseases, and become the most common chronic disease and the leading cause of morbidity. With aging, its prevalence keeps increasing. Without effective treatment, OA may eventually cause complete joint destruction, and at which time points, total joint arthroplasty is the only treatment of choice. Joint pain, swelling, and impaired functions are the common clinical manifestations of OA (Jeon et al., 2018). Joints can be considered as an organ and OA may affect all components of the organ. While central pathological change is degradation of articular cartilage, other lesions may also exist, including synovial inflammation, subchondral abnormality, meniscal damage, osteophyte formation, etc. (McCulloch et al., 2017; Yao et al., 2021). Recent studies suggest that cell senescence, synovial inflammation and epigenetic alterations are closely related to OA development (Hou et al., 2018; Zhang et al., 2019). As the pathognomonics is not completely understood, no disease modifying anti-osteoarthritis drugs (DMOADs) are not currently available. Major treatments are palliative and these include non-steroidal anti-inflammatory drugs (NSAIDs) (Crofford, 2013), intra-articular injection of hyaluronates or corticosteroids (Bannuru et al., 2019), etc. Total joint arthroplasty is only performed for the end-stage OA (Glyn-Jones et al., 2015). Clearly, OA treatment remains an unmet medical need. The development of effective and safe DMOADs depends heavily on improved understanding of OA mechanisms.

Aging is pivotal for OA pathogenesis although interdependent relationship with other risk factors may exist, including obesity, joint damage, and genetic susceptibility, etc. (Blagojevic et al., 2010; Zhang and Jordan, 2010). Aging is correlated with the number of senescent articular chondrocytes and OA progression (Price et al., 2002), suggesting that targeting senescent cells (SnCs) may prevent or reverse OA processes. In fact, a few anti-cellular senescence agents have been tested and the results are promising. These agents include sirtuin-activating compounds (STACs), senolytics, and senomorphics. Several preclinical studies have shown that these agents may delay OA progress and extend healthy lifespan. Based on these findings, clinical trials are conducted to evaluate clinical efficacy, safety profile, and side effects of these drugs.

This review provides an outline of the relationship between articular chondrocyte senescence and OA, summarizes some anti-cellular senescence strategies, and discusses future directions for potential applications in clinic.

## Aging and Cellular Senescence

Aging is a critical factor for different age-related diseases. Multiple mechanisms have been demonstrated and these include cellular senescence, telomere attrition, mitochondrial dysfunction, stem cell exhaustion, genomic instability, altered intercellular communication, epigenetic alterations, loss of proteostasis, and deregulated nutrient sensing (López-Otín et al., 2013). These processes may be interdependent and some cross-talks may exist. Cellular senescence has attracted great attention as potential therapeutics may be developed after elucidating the detailed mechanisms responsible the pathogenesis of various diseases during aging (Khosla et al., 2020).

### The Types of Cellular Senescence

The concept of “cellular senescence” was first proposed in 1961 to describe the process of irreversible growth arrest (Hayflick, 1961). It is also referred to as replicative senescence as it is induced by telomere shortening. Telomere attrition due to continuous cell division in the context of limited *in-situ* replication capacity eventually leads to this phenomenon. Replicative senescence is an important cell process to prevent carcinogenesis (Greene and Loeser, 2015). Subsequent studies show that in addition to this intrinsic pathway, there is an exogenous pathway called stress-induced premature senescence (Toh et al., 2016). Exogenous senescence results from a variety of internal and external stresses such as DNA damage, oxidative stress, oncogene activation, ultraviolet radiation, chronic inflammation and abnormal mechanical stress (Loeser, 2009; McCulloch et al., 2017). These stressors mainly activate P53/P21^CIP1^ and P16^INK4A^ tumor suppressor signaling pathways, leading to premature cell cycle arrest (Campisi and d’Adda di Fagagna, 2007; Khosla et al., 2018). Both p21^CIP1^ and p16^INK4A^ are the cyclin-dependent kinases (CDKI). P21^CIP1^ blocks CDK2-mediated pRB inactivation, while p16^INK4A^ prevents CDK4 and CDK6 mediated pRB inactivation, thereby maintaining pRB in an active form and preventing cell cycle progression (Childs et al., 2015). These two mechanisms can act independently or interact with each other, depending on cell types (Childs et al., 2015). Thus, cellular senescence is the result of the action of replicative senescence and/or stress-induced premature senescence. Currently, the molecular mechanisms of cellular senescence are still not completely understood although it is closely related to telomere erosion, DNA damage, oxidative stress, and inflammation (Toh et al., 2016).

### Characteristics of Senescent Cells

**FIGURE 1:**
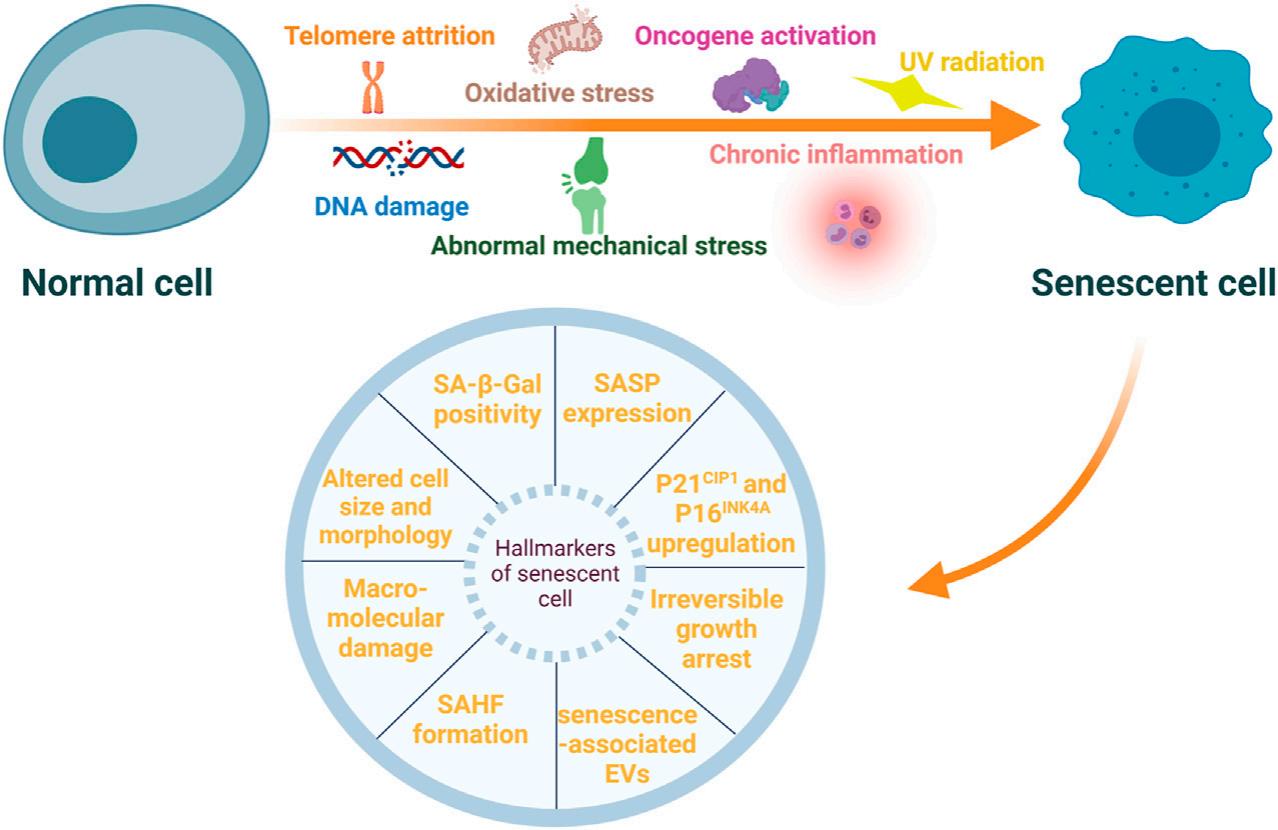
**Stressors inducing cellular senescence and hallmarkers of senescent cells.** Cellular senescence can be induced by multiple internal and external stressors, such as telomere attrition, DNA damage, oxidative stress, oncogene activation, ultraviolet (UV) radiation, chronic inflammation, and abnormal mechanical stress. These senescent cells (SnCs) exhibit a variety of typical hallmarkers, including irreversible growth arrest, altered cell size and morphology, macromolecular damage, increased expression of p21^CIP1^ and p16^INK4A^, enhanced senescence-associated-β-galactosidase (SA-β-Gal) activity, senescence-associated heterochromatic foci (SAHFs) formation, senescence-associated secretory phenotype (SASP) and extracellular vesicles (EVs) associated with senescence.

Senescent cells (SnCs) have several characteristic features such as irreversible growth arrest, altered cell size and morphology, macromolecular damage, deregulated gene expression (Campisi and d’Adda di Fagagna, 2007; Gorgoulis et al., 2019; von Kobbe, 2019), and upregulation of senescent cell anti-apoptotic pathways (SCAPs) (Kirkland and Tchkonia, 2017). Specific markers are used to identify SnCs, and these include increased expression of p21^CIP1^ and p16^INK4A^, enhanced senescence-associated-β-galactosidase (SA-β-Gal) activity, and formation of senescence-associated heterochromatic foci (SAHFs) (Sun et al., 2018). Secretion of extracellular vesicles, including exosomes and microvesicles, is also upregulated in SnCs (Effenberger et al., 2014; Fafián-Labora and O’Loghlen, 2021). These senescent-associated extracellular vesicles may also induce senescence in adjacent non-senescent cells (Jeon et al., 2019). Most importantly, SnCs exhibit altered secretory characteristics and can rapidly secrete a variety of inflammatory cytokines, growth factors, chemokine, soluble and insoluble factors and matrix metalloproteinases (MMPs). These cells are referred to as senescence-associated secretory phenotype (SASP) (Sun et al., 2018). The formation of SASP is mainly mediated by nuclear factor-κB (NF-κB) pathway (Salminen et al., 2012), and the components of SASP may differ in different types of stresses and cells undergoing senescence (Birch and Gil, 2020). By autocrine or paracrine mechanisms, SASP factors enhance and prolong the senescent state (Muñoz-Espín and Serrano, 2014; Sun et al., 2018). It cannot over-emphasize that SASP factors may affect adjacent non-SnCs by activating various cell surface receptors and corresponding signal transduction pathways, forming a circuit of SnCs and microenvironmental communication (Coppé et al., 2010). Moreover, persistent existence of the SASP factors incites inflamm-aging (Shetty et al., 2018), a chronic, systemic, low-grade inflammation state during aging process. Inflamm-aging is another major feature of the aging process and can be caused by sustained antigenic load and stress (Franceschi et al., 2000). It may result from abnormal functions of innate and adaptive immune systems (Prieto et al., 2020). Of note, SASP may have beneficial effects, for example, SnCs may sense tissue damage and initiate tissue repair through the SASP effect. However, persistent SASP can disrupt normal tissue structures and functions (Coppé et al., 2008). Even if the number of SnCs is small, they can interrupt normal functions of the surrounding cells via SASP factors, cause microenvironment dysfunction, and incite the pathogenesis of some age-associated diseases (Sun et al., 2018; Kim and Kim, 2019). Stressors inducing cellular senescence and hallmarkers of SnCs are presented in Figure 1.

However, these features are not specific to SnCs, and not all SnCs express these markers. So far, universal markers expressed only in SnCs have not been identified, thus, multiple markers are needed to identify SnCs (Campisi, 2011; McHugh and Gil, 2018). Improved reliability and accuracy of detecting SnCs may be achieved by using different senescent markers including SA-β-Gal, p21^CIP1^, p16^INK4A^, SASP, and DNA damage (Gorgoulis et al., 2019).

Cellular senescence is an example of antagonistic pleiotropy (Giaimo and d’Adda di Fagagna, 2012). In early acute senescence, low level of SnCs has a positive effect in human biological processes including tumor suppression, wound healing, embryonic development, tissue regeneration and remodeling. However, long-term macromolecular damage can cause chronic senescence due to decreased clearance of SnCs by the immune system with aging (Childs et al., 2015; Ovadya et al., 2018). These continuously accumulated SnCs have deleterious effects (van Deursen, 2014; von Kobbe, 2019). A study shows that SnCs can propagate DNA damage and induce senescence in their neighboring cells through a paracrine mechanism (Nelson et al., 2012), leading to amplification of pathological processes and pathogenesis of the age-related diseases including OA.

## Senescence of Articular Chondrocytes in OA

The age-related changes in OA affect all components of a joint including articular cartilage, synovium, muscles, and ligaments (Zhang et al., 2019). Aging and OA are closely related although OA can occur in the absence of significant aging (Loeser, 2009). For example, SnCs have been found in young post-traumatic OA patients (McCulloch et al., 2017). SnCs and SASP factors are detected in OA tissues including articular cartilage, subchondral bone, synovium, and infrapatellar fat pad (McCulloch et al., 2017; Jeon et al., 2018). Degradation of articular cartilage is the central pathology of OA, and articular chondrocytes are the main resident cell type in hyaline cartilage, although emerging data indicate that chondroprogenitor cells may also exist. This is why articular chondrocytes are extensively studied (Vinod et al., 2018; Zhang et al., 2019; Carluccio et al., 2020; Hu et al., 2021). Chondrocytes produce extracellular matrix (ECM), mainly type II collagen (Col-II) and proteoglycan including aggrecan (McCulloch et al., 2017). Together with other EMC components, they maintain the structure and function of articular cartilage. Senescent chondrocytes were found in hip and knee cartilage in OA patients (Price et al., 2002). Generally, articular chondrocytes are quiescent cells with very limited proliferative capacity (Loeser et al., 2012), and little or no replicative senescence occurs (Mobasheri et al., 2015). The length of telomere in these cells gradually shortens with age (Martin and Buckwalter, 2001), and this non-replicative telomere erosion indicate that most of the senescent chondrocytes in OA are induced by endogenous and exogenous stress. Chondrocyte senescence impairs cartilage homeostasis, leading to cartilage degeneration in isolated human articular cartilage chondrocytes from donors ranging in age from 1 to 87 years (Martin and Buckwalter, 2003). Interestingly, otherwise quiescent chondrocytes become “activated” in OA with formation of chondrocyte clusters and enhanced production of different cytokines (Goldring and Marcu, 2009). Senescent chondrocytes are less responsive to anabolic cytokines, while they are more sensitive to catabolic, proinflammatory ones (Zhou et al., 2004), which may exacerbate the catabolic inflammatory environment, indirectly reduce the repair capacity of articular cartilage and contribute to OA progression.

**FIGURE 2:**
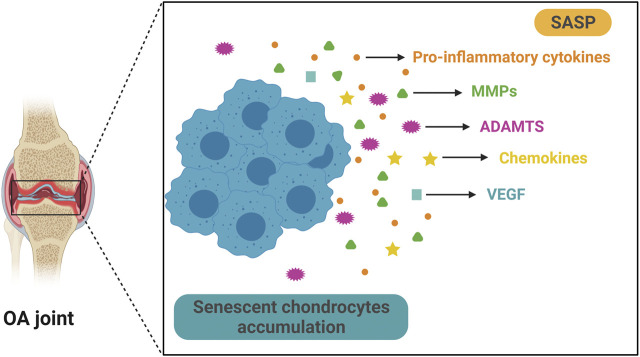
**Senescent chondrocytes accumulate and secrete SASP in OA.** Aging is significantly associated with OA and senescent articular chondrocytes play a key role in OA progression. Senescent chondrocytes exist and accumulate in OA joint and they secrete multiple SASPs, such as pro-inflammatory cytokine, matrix metalloproteinases (MMPs), a disintegrin and metalloproteinase with thrombospondin motifs (ADAMTS), chemokine and vascular endothelial growth factor (VEGF). These SASPs can cause articular cartilage damage and exacerbate pathological changes in OA.

Similar to other types of SnCs, OA chondrocytes exhibit specific senescent markers such as p16^INK4A^ (Zhou et al., 2004), increased SA-β-Gal activity, etc. (Rose et al., 2012). In articular cartilage samples from OA patients, the activity of SA-β-Gal correlates with the OA severity (Gao et al., 2016). Importantly, some SASP factors are also present in OA chondrocytes. These include pro-inflammatory cytokines such as IL-1β, IL-6, and TNF-α, matrix degradation enzymes such as MMPs and ADAMTS4/5, chemokine such as CCL2 and MCP-1 and growth factors such as VEGFs (Vinatier et al., 2018) (Figure 2). These factors may cause degradation of ECMs in articular cartilage, which could then amplify synovial inflammation (Sellam and Berenbaum, 2010). Synovial inflammation increases production of MMP-13, and accumulation of matrix debris further incites synovial inflammation, forming a vicious cycle, which eventually leads to total joint destruction. TNF-α, IL-1β, and IL-6 are among the most important proinflammatory cytokines in synovial inflammation and cartilage destruction (Kapoor et al., 2011). These cytokines also induce the formation of SASP in senescent chondrocytes. IL-1β significantly increases the expression of other proinflammatory cytokines and MMPs in human chondrocytes (Aida et al., 2005; Aida et al., 2006). TNF-α upregulates the expression of MMPs and ADAMTS, causing further degradation of cartilage matrix (Wang et al., 2019). Synovium also contributes to the senescence burden in knee joints during OA development (Jeon et al., 2017). SASP levels are significantly upregulated in isolated human OA patient synovial tissue, and the SASPs may cause or exacerbate synovial inflammation (Zhang Y. et al., 2020). In addition, extracellular vesicles secreted by senescent OA chondrocytes induce senescence-like phenotypes in adjacent non-SnCs and inhibit chondrogenesis via a para-senescent effect (Jeon et al., 2019).

Several lines of evidence suggest a critical role of cellular senescence in OA formation, and great endeavors have been made to dissect molecular mechanisms in these processes. Senescent cells from ear fibrocartilage transplanted to mouse knee joints results in hindlimb pain, impaired mobility, and OA-like changes such as articular cartilage damage, osteophyte formation, altered subchondral bone structure and meniscus injury (Xu et al., 2017), suggesting a causative effect of cellular senescence on OA. To confirm these findings, the p16-3MR transgenic mice were generated and a post-traumatic OA model was established. SnCs were detected in articular cartilage and synovium in these mice. Selective removal of SnCs expressing p16^INK4A^ after injection of ganciclovir ameliorated joint pain and downregulated the expression of MMP-13 and IL-1β. Meanwhile, cartilage regeneration was enhanced (Jeon et al., 2017). These findings suggest that senescent chondrocytes play an important role in OA development, and that anti-cellular senescence strategies may be a promising approach to prevent or cure OA. In this review, we focused on chondrocyte senescence and the anti-cellular senescence treatments mainly targeting chondrocytes. The agents targeting other types of joint cells will be reviewed in other papers.

## Anti-Cellular Senescence Strategies

**FIGURE 3:**
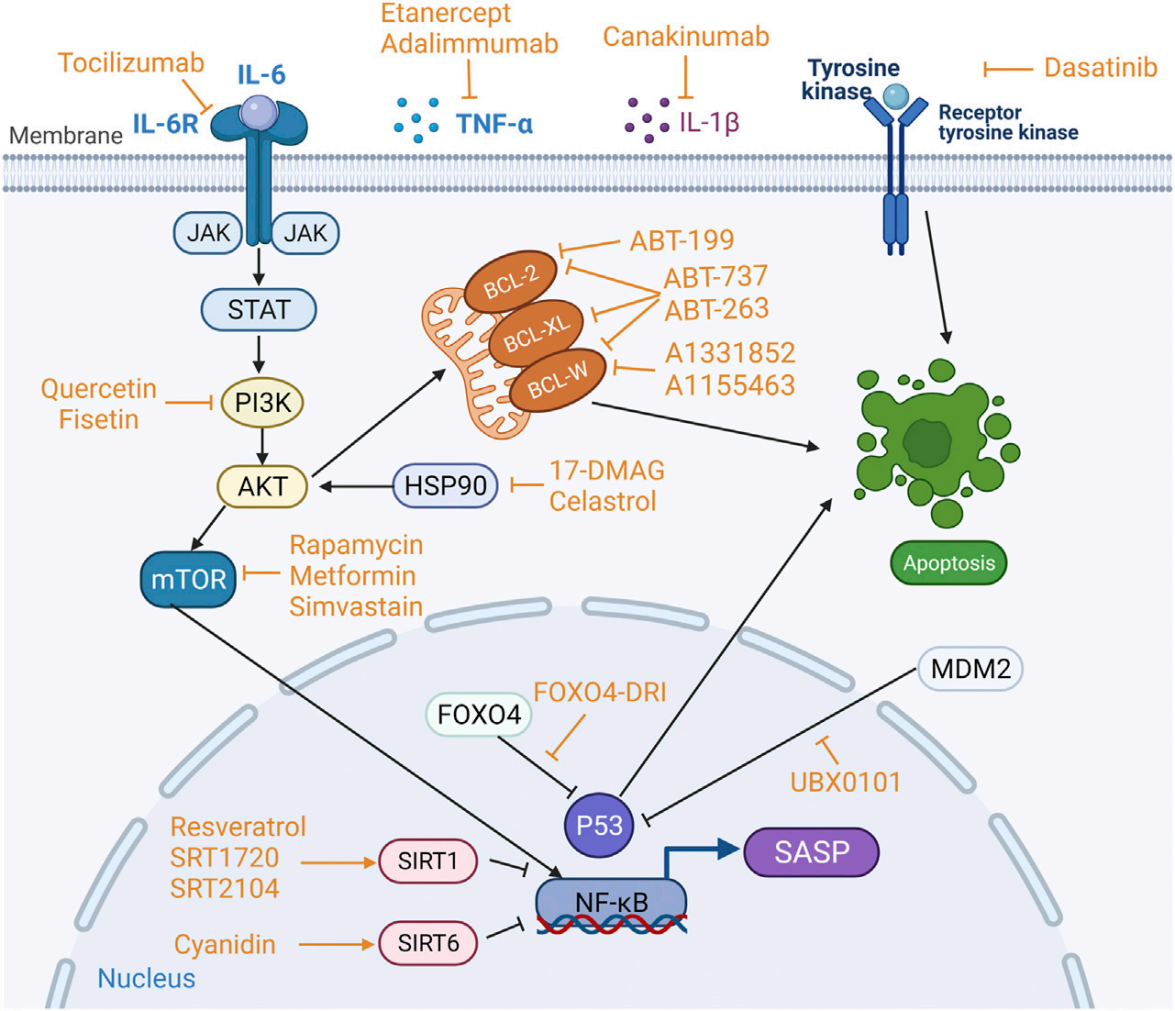
**Targets of sirtuin-activating compounds (STACs), senolytics and senomorphics.** Anti-apoptotic pathways and pro-survival pathways are upregulated in senescent cells (SnCs), and the key proteins involved in the anti-apoptotic pathway are BCL-2, BCL-W and BCL-XL in the BCL-2 protein family, and p53 phosphatidylinositol 3-kinase (PI3K), AKT, FOXO4, tyrosine kinase and heat shock protein 90 (HSP90). Different anti-cellular senescence agents act on different targets. STACs resist aging mainly by activating sirtuins. Senolytics target anti-apoptotic pathways to induce apoptosis in SnCs. Senomophics suppress SASP function by inhibiting the production of SASP or neutralizing a specific SASP component, thereby inhibiting cellular senescence.

A variety of anti-cellular senescence agents, namely, STACs, senolytics, and senomorphics, have been discovered and developed. These agents take their effect either by inducing apoptosis of SnCs and or suppressing harmful activity of the SASP factors (Figure 3). Observations made in some preclinical experiments are promising, which may facilitate the development of novel therapeutics with the DMOAD properties.

### Sirtuins and Sirtuin-Activating Compounds

The sirtuins family are highly conserved NAD^+^-dependent histone and non-histone deacetylases that regulate cellular senescence, DNA repair, inflammatory response, metabolism, apoptosis and cell survival (Michan and Sinclair, 2007). The first sirtuin, e.g., Sir2, was found in *saccharomyces cerevisiae* and able to extend their lifespan (Imai et al., 2000; Michan and Sinclair, 2007). Currently, there are seven known Sir2 homologs in mammals, referred to as SIRT1-SIRT7 (Frye, 2000). SIRT1 and SIRT6 are closely related to OA formation.

The targets of SIRT1 are histones and non-histone proteins. Non-histone proteins include transcription factors such as FOXO (Brunet et al., 2004), p53 (Ong and Ramasamy, 2018) and NF-κB (Yeung et al., 2004; Nakagawa and Guarente, 2014). Previous reports indicate that autophagy declines with aging. Autophagy maintains cellular homeostasis and function, while the loss of autophagy leads to mitochondrial dysfunction and dysregulation of protein homeostasis that may exacerbate senescence (Leidal et al., 2018; Vinatier et al., 2018). With aging, autophagy in chondrocytes gradually decreases, which may be related to aging-related OA progression in mice (Caramés et al., 2010). Another study confirms that autophagy deficiency might induce chondrocyte senescence and cartilage destruction, which may contribute to OA development in mice (Caramés et al., 2015). SIRT1 enhances autophagy directly or by inhibiting NF-κB signaling (Salminen and Kaarniranta, 2009). It can also attenuate the senescence-related inflammation by deacetylating histones and the transcription factor NF-κB (Xie et al., 2013). In addition, inhibiting NF-κB pathway may induce activation of stem cells in articular cartilage, leading to cartilage regeneration in rat OA (Tong et al., 2015). FOXO plays an important role in stress resistance of chondrocytes and maintenance of cartilage homeostasis, and SIRT1 help maintain joint integrity by regulating FOXO (Akasaki et al., 2014; Matsuzaki et al., 2018; Lee et al., 2020). Cartilage samples from patients with OA show downregulated expression of SIRT1 in articular chondrocytes (Fujita et al., 2011). Knock-out of *SIRT1* gene enhances the expression of MMP-13 in mouse articular chondrocytes, leading to excessive degradation of cartilage matrix and aggravation of OA lesions (Matsuzaki et al., 2014; Elayyan et al., 2017). These studies suggest that SIRT1 has cartilage-protective effect, and that enhancing its activity may prevent OA progression.

SIRT6 is an important factor in the aging process. It regulates tissue homeostasis, maintains genome stability, prevents DNA damage, and preserves normal redox status in human cartilage during aging (Collins et al., 2021). Overexpressing SIRT6 in mice inhibits the senescence of articular chondrocyte and joint inflammation mediated by NF-κB signaling pathway (Wu et al., 2015). Consistently, knock-out SIRT6 in human chondrocytes results in upregulation of MMP-3 and MMP-13 expression, increased DNA damage and telomere dysfunction, leading to chondrocyte senescence (Nagai et al., 2015). Further, in articular cartilage form OA patients, SIRT6 expression is significantly reduced (Wu et al., 2015). Collectively, these observations suggest that modulation of SIRT6 activities may change senescent process of articular chondrocytes and prevent OA development. Accordingly, intensive studies have been conducted to evaluate the effect of sirtuin-activating compounds (STACs) on OA. Previous reports show that STACs for SIRT1 have anti-aging and chondro-protective effects and these STACs share a common allosteric activation mechanism (Hubbard and Sinclair, 2014; Sinclair and Guarente, 2014). The first natural activator of SIRT1 was polyphenols from plants. For example, resveratrol may extend the lifespan of *saccharomyces cerevisiae* and activate SIRT1 with subsequent deacetylation and inhibition of p53 pathway, promoting cell survival (Howitz et al., 2003).

Calorie restriction is the only non-genetic method to extend the maximum lifespan in mammals (Michan and Sinclair, 2007). Resveratrol mediated anti-aging effect of calorie restriction activates SIRT1, which regulates the longevity pathways and related factors including increased insulin sensitivity, decreased IGF-I expression, increased AMP-activated protein kinase (AMPK) activity, and increased number of mitochondria (Baur et al., 2006). In addition, resveratrol inhibits MMP-13 expression in human OA chondrocytes after activation of SIRT1 (Elayyan et al., 2017). Further, it effectively prevents destruction of articular cartilage in OA mice by inhibiting NF-κB and HIF-2α pathway in a SIRT1-dependent mechanism (Li et al., 2015). Preclinical studies have demonstrated that resveratrol significantly lowers the serum levels of IL-1β, IL-6, and TNF-α and effectively reduces the severity of pain and stiffness. Therefore, it can improve knee joint function in OA patients and exhibit excellent safety profile and tolerability (Hussain et al., 2018; Marouf et al., 2018). Cyanidin, a SIRT6 activator, is able to upregulate SIRT6 expression in a dose-dependent manner. It significantly decreases IL-1β-induced expression of TNF-α, IL-6, COX-2, MMP-13, and ADAMTS-5, thereby preventing synovial inflammation and cartilage degradation. In articular cartilage of OA mice, cyaniding downregulates the expression of MMP-13 and upregulates the expression of Col-II, preventing matrix degradation in articular cartilage (Jiang et al., 2019).

To improve the efficacy and specificity of STACs, researchers have focused on synthetic STACs. After high-throughput screening, several promising agents have been identified, including SRT1460, SRT1720, SRT2183 (Milne et al., 2007), SRT2104 (Libri et al., 2012), and SRT3025 (Artsi et al., 2014). Different scaffolds have been tested such as imidazolothiazole, oxazolopyridine, benzimidazole, azabendazole (Bemis et al., 2009), and urea-based scaffolds (Dai et al., 2010). Compared to natural STACs, synthetic STACs have higher potency, solubility, specificity, and bioavailability (Sinclair and Guarente, 2014).

SRT1720, a potent SIRT1 activator, can significantly decrease the expression of MMP-13 and ADAMTS-5 in chondrocytes, reduce articular cartilage degeneration. Besides, it inhibits osteophyte formation and attenuates synovial inflammation. As such, it delays OA progression in mice (Nishida et al., 2018). Moreover, SRT1720 can prolong the lifespan and improve health status of mice (Mitchell et al., 2014). SRT2104 is a novel STAC with high selectivity for SIRT1 and able to prevent the progression of knee OA in mice. It can reduce the levels of MMP-13, ADAMTS-5, IL-1β, IL-6 and acetylated NF-κB p65, and increase Col-II synthesis in cartilage (Miyaji et al., 2020). Similar to SRT1720, SRT2104 also improves systemic physiology and prolongs healthy lifespan of mice. Its anti-inflammatory and antioxidant effects are related to reduced NF-κB activity (Mercken et al., 2014). Non-therapeutic clinical studies show that SRT2104 has excellent safe profile and tolerability (Libri et al., 2012; Hoffmann et al., 2013). Collectively, these studies demonstrate the potential of STACs for the treatment of OA, and preclinical studies and clinical trials may be conducted to confirm their efficacy and safety profile. Preclinical and clinical studies are summarized in Table 1.

**TABLE 1:** STACs on OA treatment.

STACs	Targets	Preclinical and clinical research of OA and others
Resveratrol	SIRT1	Prevented articular cartilage damage in OA mice Li et al. (2015); Alleviated pain and stiffness in knee OA patients Hussain et al. (2018); Marouf et al. (2018).
SRT1720	SIRT1	Delayed OA progression in mice Nishida et al. (2018).
SRT2104	SIRT1	Delayed knee OA progression in mice Miyaji et al. (2020).
Cyanidin	SIRT6	Inhibited degradation of ECM in human OA chondrocytes *in vitro* and delayed OA progression in mice Jiang et al. (2019).

### Senolytics

As mentioned above, the senescence of articular chondrocyte plays a key role in the development and progression of OA, and accumulation of senescent chondrocytes accelerates the formation of OA. Senolytics are a novel class of anti-senescence drugs that can target and kill SnCs. Anti-apoptotic and pro-survival pathways are upregulated in SnCs (Zhu et al., 2015). Multiple factors are involved in this pathophysiological process such as BCL-2, BCL-W, and BCL-XL in the BCL-2 protein family, and p53, p21^CIP1^, phosphatidylinositol 3-kinase (PI3K), AKT, FOXO4, tyrosine kinase, and heat shock protein 90 (HSP90) (von Kobbe, 2019; Martel et al., 2020). Pro-survival pathways can protect SnCs from their own SASP effect (Romashkan et al., 2021), and these survival networks mainly include PI3K-AKT pathway, BCL-2/BCL-Xl pathway, p53/p21^CIP1^, different kinases, etc. (Zhu et al., 2017). Therefore, a variety of senolytics have emerged to target anti-apoptotic and pro-survival pathways of SnCs, and these include kinase inhibitors, BCL-2-family protein inhibitors, natural compounds, FOXO4-DRI peptides, HSP90 inhibitor, UBX0101 (Niedernhofer and Robbins, 2018), and galactose-encapsulated cytotoxic drugs that specifically kill SnCs (Muñoz-Espín et al., 2018).

In 2015, the first senolytics, dasatinib (a tyrosine kinase inhibitor) (Montero et al., 2011) and quercetin (a flavonoid with antioxidant and estrogenic activities that inhibit PI3K and other kinases), were demonstrated to be able to specifically kill SnCs. The combination of these two drugs may eliminate different types of SnCs, reduce the burden of SnCs in aged mice, significantly enhance body function and prolong healthy lifespan (Zhu et al., 2015; Xu et al., 2018). In skeletal tissues, such a combination may remove senescent osteocytes, reduce age-related bone loss, and improve the microarchitecture of both trabecular and cortical bone in aged mice (Farr et al., 2017). In addition, they can selectively induce apoptosis of senescent chondrogenic progenitor cells and ameliorate the harmful effect of SASP, therefore, promoting chondrogenesis and cartilage regeneration in rat OA (Dai et al., 2020). A clinical trial shows that dasatinib together with quercetin can improve lung function in patients with idiopathic pulmonary fibrosis caused by senescence of lung cells through their anti-senescence effect (Justice et al., 2019). Preliminary results from a phase I clinical trial to treat diabetic patients with chronic kidney disease using these two drugs reduces SnCs expressing p16^INK4A^ and p21^CIP1^ in adipose and epidermal tissues, and decreases the critical SASP factors levels in plasma, implying senolytics could reduce the burden of SnCs in the human (Hickson et al., 2019). However, their effect on OA remains unknown.

Fisetin, a member of the flavonoid family, is a naturally polyphenolic compound with antioxidant and anti-inflammatory properties (Khan et al., 2013). It has similar mechanism to quercetin in that it may take effect through PI3K/AKT/mTOR and NF-κB pathways (Myrianthopoulos, 2018). It significantly downregulates the expression of p16^INK4A^ and effectively removes SnCs in premature aging mice. It also prolongs the healthy lifespan of aged mice and reduces age-related pathological changes (Yousefzadeh et al., 2018). Meanwhile, fisetin also significantly decreases IL-1β-induced expression levels of IL-6, TNF-α, MMP-3, MMP-13, ADAMTS-5, prevents degradation of aggrecan and Col-II in human OA cartilage by activating SIRT1. In OA mice, fisetin prevents cartilage destruction and synovial inflammation (Zheng et al., 2017). Clinical trial of fisetin for the treatment of OA is under way.

Piperlongumine, a natural senolytics, is able to selectively induce apoptosis in senescent humans WI38 fibroblast (Wang et al., 2016; Zhang et al., 2018). Its working mechanism is not clear although it may induce proteasomal degradation of OXR1, an important antioxidant protein regulating the expression of multiple antioxidant enzymes (Zhang et al., 2018). Interestingly, piperlongumine also exhibits senomorphic activity in that it significantly reduces serum levels of IL-1β, MMP-1, MMP-3 in OA rats (Ye et al., 2020).

BCL-2 family protein inhibitors demonstrate senolytic properties via inhibition of anti-apoptotic proteins such as BCL-2, BCL-Xl, and BCL-W in SnCs. Small molecule inhibitor ABT-737 may specifically induce apoptosis of different SnCs by repressing anti-apoptotic proteins BCL-2, BCL-Xl, and BCL-W. This study also shows that BCL-W and BCL-XL are more important with regards to anti-apoptotic property of SnCs compared BCL-2 (Yosef et al., 2016). However, ABT-737 is not orally bioavailable, which would limit flexibility. Based on the pharmacological properties of ABT-737, navitoclax (ABT-263) is a second-generation, orally bioavailable small-molecule Bcl-2 family protein inhibitor which also targets BCL-2, BCL-XL, and BCL-W (Tse et al., 2008). It selectively eliminates senescent hematopoietic stem cells from bone marrow and muscle stem cells in aged mice through inhibition of anti-apoptotic pathway (Chang et al., 2016). A recent study showed that navitoclax may effectively remove accumulated senescent chondrocytes by inducing cell apoptosis in isolated human OA articular cartilage tissues. In addition, navitoclax downregulated the expression of SASP-related factors and increased chondrogenic phenotype, alleviated inflammation, prevented cartilage matrix degradation, and enhanced ECM deposition. Intra-articular injection of ABT-263 prevented the pathological changes in articular cartilage and subchondral bone in OA rats (Yang et al., 2020). However, severe adverse effect such as thrombocytopenia prevents its widespread use (Cang et al., 2015). In contrast, the selective BCL-2 inhibitor ABT-199, and the BCL-XL inhibitors A1331852 and A1155463, exhibits less hematotoxicity compared to ABT-263 (Cang et al., 2015; Leverson et al., 2015).

UBX0101, an inhibitor of protein interaction between MDM2 and p53, exhibits senolytic activity through p53-mediated clearance of SnCs (Thoppil and Riabowol, 2019). It induces apoptosis of senescent chondrocytes from human OA cartilage. In the OA mice, UBX0101 effectively removes SnCs from articular cartilage by enhancing apoptotic pathways, reduces the expression levels of p16^INK4A^, p21^CIP1^, IL-1β, and MMP-13, alleviates joint pain, reduces destruction of articular cartilage, and promotes cartilage regeneration. Even in the late stages, it may still lessen the OA-like pathological changes (Jeon et al., 2017). The results of the phase I clinical trial showed that UBX0101 reduced joint pain and improved function in OA patients with satisfactory safety and tolerability (Hsu et al., 2020). However, a phase II clinical trial failed to confirm the properties of UBX0101 in OA. (https://clinicaltrials.gov/ct2/show/NCT04349956). Similarly, the FOXO4-DRI peptide interferes with FOXO4 interaction with p53. It also specifically eliminates SnCs by inducing p53-dependent apoptosis and restores tissue homeostasis in aged mice (Baar et al., 2017).

Heat shock protein 90 (HSP90) inhibitors may become potential senolytics. HSP90 is a highly conserved protein involved in a variety of physiological and pathological processes (Taipale et al., 2010). Although there is no significant difference in the expression level of HSP90 between senescent and non-senescent cells, its downstream molecule AKT is upregulated in SnCs (Thoppil and Riabowol, 2019). HSP90 inhibitors can dephosphorylate and block interaction between HSP90 and phosphorylated AKT, interrupting PI3K/AKT pathway and inducing the apoptosis of SnCs (Fuhrmann-Stroissnigg et al., 2017). 17-DMAG (Alvespimycin), a geldanamycin-derived HSP90 inhibitor, can selectively kill SnCs without affecting healthy cells, thus reducing the age-related symptoms and extending the lifespan of Ercc1^–/Δ^ premature senescent mice (Fuhrmann-Stroissnigg et al., 2017). Meanwhile, 17-DMAG downregulates the expression of p21^CIP1^. It promotes fibrocartilage formation in mouse ears (Bertram et al., 2018), whereas its role in articular cartilage remains unknown.

Celastrol, another HSP90 inhibitor, can prevent IL-1β-induced activation of NF-κB signaling pathway and upregulate the expression of MMPs, resulting in reduced catabolism and attenuated inflammatory responses in human OA chondrocytes (Ding et al., 2013). Data from a study of transcriptomics and network pharmacology suggest that celastrol may target multiple genes involved in cell senescence in OA including TP53, mTOR, mitogen-activated protein kinase (MAPK) 1, STAT3, and others (Dai et al., 2021). Due to the low solubility, hollow mesoporous silica nanoparticles were used to deliver celastrol, and the results showed that such system can improve the bioavailability of celastrol. After intra-articular injection, such combination significantly reduces the production of inflammatory factors, prevents the destruction of articular cartilage, and improves the therapeutic effect of rat OA (Jin et al., 2020).

Peroxisome associated-activated receptor-α (PPARα) agonists are FDA-approved drugs regulating lipid metabolism. A recent study shows that fenofibrate, a PPARα agonist, has senolytic activity. It induces apoptosis of senescent human OA chondrocytes and enhances cell autophagy, resulting in reduced inflammation and cartilage degradation, alleviation of joint pain and preservation of joint structures in OA patients (Nogueira-Recalde et al., 2019). More trials need to be conducted before its wide use in clinic.

Senolytics appear to be cell type-specific (Y et al., 2017). One explanation is that senescence is involved in multiple pathways and proteins that may differ in different cell types and the sensitivities to senolytics may vary in different SnCs. Thus, it is unlikely that a single senolytic drug will eliminate all types of SnCs. For example, dasatinib is more potent in elimination of senescent human pre-adipocytes, while quercetin is more specific for senescent human vein endothelial cells, human endothelial cells and mouse bone marrow mesenchymal stem cells. The combination of dasatinib and quercetin significantly reduces the number of mouse embryonic fibroblasts (Zhu et al., 2015).

A new approach has been developed in order to improve the specificity of senolytics to certain types of SnCs. The oligo-galactose was used as a delivery system to carry different senolytics drugs. It takes advantage of the increased lysosomal *β*-galactosidase activity in SnCs and high affinity of galactose to SnCs for targeted elimination. For example, the encapsulation of cytotoxic drug adriamycin in oligo-galactose nanoparticles can selectively kill SnCs in aged mice through endocytosis. Such system also reduces organ damages due to off-target effect (Muñoz-Espín et al., 2018). Galactose-conjugation of navitoclax (Nav-Gal) is used as a prodrug with senolytic property, which can be preferentially activated by enhanced SA-β-Gal activity in SnCs, leading to targeted release into SnCs to induce their apoptosis. Compared with navitoclax, Nav-Gal has higher specificity and lower hematological toxicity (González-Gualda et al., 2020). SSK1 is another promising prodrug. In aged mice, SSK1 is specifically cleaved in SnCs and gemcitabine is released, leading to activation of MAPK pathway and apoptosis of SnCs. It also reduced SASP levels, thereby alleviating senescence-related detrimental effects, downregulating inflammatory responses and restoring motor function (Cai et al., 2020). This strategy paves a new way for OA treatment. Preclinical and clinical studies are summarized in Table 2.

**TABLE 2:** Senolytics on anti-aging therapy.

Senolytics	Targets	Preclinical and clinical research of OA and others
Dasatinib	Tyrosine kinase	The combination of dasatinib and quercetin reduced age-related bone loss Farr et al. (2017).
		Improved articular cartilage integrity in post-traumatic OA rats Dai et al. (2020).
		Showed promising effects in clinical trials in idiopathic pulmonary fibrosis patients and diabetic nephropathy patients Hickson et al. (2019); Justice et al. (2019).
Quercetin	PI3K	As above
Fisetin	PI3K/AKT/mTOR; SIRT1; NF-κB	Reduced IL-1β-induced inflammatory response and human chondrocyte ECM degradation; delayed OA progression in mice Zheng et al. (2017).
		The clinical trial of fisetin for the treatment of OA is recruiting (https://clinicaltrials.gov/ct2/show/NCT04210986).
Piperlongumin	OXR1	Protected articular cartilage in OA rats Ye et al. (2020).
ABT-737	BCL-2; BCL-XL; BCL-W	Induced apoptosis of multiple senescent cells Yosef et al. (2016).
ABT-263 (Navitoclax)	BCL-2; BCL-XL; BCL-W	Induced apoptosis of senescent chondrocytes isolated human OA articular cartilage and protected articular cartilage in OA rats Yang et al. (2020).
A1331852	BCL-XL	Killed several tumor cells Leverson et al. (2015).
A1155463	BCL-XL	Killed several tumor cells Leverson et al. (2015).
ABT-199 (Venetoclax)	BCL-2	FDA approved for chronic lymphocytic leukemia Cang et al. (2015).
UBX0101	MDM2/p53	Induced apoptosis of senescent chondrocytes isolated from cartilage of OA patients and reduced erosion of articular cartilage in OA mice Jeon et al. (2017).
		Reduced joint pain of OA patients in the phase I clinical trial Hsu et al. (2020).
		The phase II clinical trial failed (https://clinicaltrials.gov/ct2/show/NCT04349956).
FOXO4-DRI	FOXO4/p53	Eliminated senescent cells restored tissue homeostasis in aged mice Baar et al. (2017).
17-DMAG (Alvespimycin)	HSP90; p21	Extended the lifespan of Ercc1^-/Δ^ premature aging mouse Fuhrmann-Stroissnigg et al. (2017).
		Promoted auricular cartilage formation in the mice Bertram et al. (2018).
Celastrol	HSP90; TP53; mTOR; MAPK1; STAT3; NF-κB	Attenuated inflammatory responses in human OA chondrocytes Ding et al. (2013).
		Reduced articular cartilage destruction in OA rats Jin et al. (2020).
Fenofibrate	PPARα	Induced apoptosis of senescent human OA chondrocytes and alleviated joint pain and joint destruction in OA patients Nogueira-Recalde et al. (2019).
Gal-encapsulated cytotoxic	SA-β-Gal	Killed senescent cells specifically and reduced off-targets Muñoz-Espín et al. (2018).
Gal-encapsulated navitoclax	SA-β-Gal	Killed senescent cells specifically and reduced off-targets González-Gualda et al. (2020).

### Senomorphics

Senomorphics, another type of anti-cellular senescence drugs, can ameliorate the deleterious effects of SnCs by inhibiting SASP factors without inducing apoptosis of SnCs (Kim and Kim, 2019). SASP is primarily driven by the inflammatory transcription factor NF-κB, and other regulatory pathways including mTOR, MAPK, and JAK/STAT pathways (Soto-Gamez and Demaria, 2017; Hernandez-Segura et al., 2018). Senomorphics reduce the production of SASP factors, or neutralize specific SASP components. These properties render senomorphics the potential to become promising agents for OA treatment.

#### Senomorphics Targeting SASP Regulatory Pathways

Rapamycin, the inhibitor of a mammalian target of rapamycin (mTOR), is an FDA-approved drug primarily for rejection of transplants. It mainly affects mTORC1, which regulates major cellular processes including protein and lipid synthesis, autophagy and aging (Laplante and Sabatini, 2012). Recent studies have demonstrated that rapamycin is a potent SASP inhibitor although it is unable to reverse cellular senescence. It inhibits the expression of IL-1 by suppressing mTORC1 activity, while the reduction of IL-1α blocks the production of other SASP factors including IL-6, IL-8, CSF-2, CCL-8, and BMP-4 through inhibition of NF-κB pathway, thus, alleviating the senescence-related inflammation (Laberge et al., 2015). Alternatively, during aging, mTOR regulates the translation of MAPKAPK2, a downstream factor of p38 MAPK. It can phosphorylate ZFP36L1 and inhibit the disruption of SASP mRNA stability maintained by ZFP36L1 (Herranz et al., 2015). In addition, rapamycin may promote chondrocyte autophagy by inhibiting mTORC1, therefore, attenuating OA (Caramés et al., 2012). Another study shows that mTORC1 may inhibit chondrocytes hypertrophy in mouse OA via Wnt 16-PCP/JNK and PTHrP pathways (Tong et al., 2019). Rapamycin also reduces the numbers of p16 ^INK4A^ and p21^CIP1^ positive cells, activates autophagy pathway and inhibits cell cycle arrest (Wang et al., 2017). Besides, it inhibits IL-18-mediated production of the SASP factors such as MMP-1, MMP-13, and IL-6. Reduced inflammatory responses, suppressed cartilage degradation and enhanced autophagy prevent cartilage destruction and ameliorate rat OA lesions (Bao et al., 2020). However, rapamycin may have side effects such as diarrhea, vomiting, and vasculitis (Abraham and Wiederrecht, 1996). In order to avoid side effects, PLGA microparticles were utilized as a drug carrier to deliver rapamycin, and results showed that such system effectively inhibited chondrocyte senescence under oxidative stress and prolonged the half-life of rapamycin in joint cavity (Dhanabalan et al., 2020).

Histone deacetylase inhibitors (HDACi) repress the enzymatic activity of HDAC and promote the acetylation of proteins. These agents have been trialed for the treatment of a variety of diseases including OA. Although HDACi exhibits senolytic activity in other diseases (Samaraweera et al., 2017), it seems to have the properties of senomorphics in OA models. Both broad-spectrum HDACi trichostatin-A and selective HDACi entinostat (MS-275) may downregulate the expression of ADAMTS-5 and MMP-3 induced by mechanical stress through suppressing MAPK signaling pathway in human chondrocytes (Saito et al., 2013). Treatment with trichostatin-A significantly repressed the expression of MMPs including MMP-1, MMP-3, and MMP-13 and the pro-inflammatory cytokines including TNF-α, IL-1β, and IL-6, thereby, inhibiting matrix degradation in mouse OA cartilage. The protective effect of HDACi on articular cartilage in OA is dependent on Nrf2, a key transcription factor that can regulate antioxidant defense system and suppress inflammation (Cai et al., 2015). Some other HDACi have been trialed and these include orinostat (SAHA), ricolinostat (ACY-1215), givinostat (ITF2357), butyrateacid, and valproicacid (VPA). The results showed that they had chondroprotective effects through inhibition of the expression of MMPs and pro-inflammatory cytokines in human OA chondrocytes via NF-κB and MAPK signaling pathways (Zhang H. et al., 2020).

Scutellariae is a natural flavonoid with various biological activities, and apigenin derived from scutellariae inhibits IL-1β-induced gene expression, secretion, and enzyme activity of MMP-3 in articular chondrocytes *in vitro* and *in vivo*. In addition, Apigenin may protect articular cartilage by repressing the expression of MMP-1, MMP-13, ADAMTS-4, and ADAMTS-5 (Park et al., 2016). Furthermore, several studies have demonstrated that scutellariae exerts its cartilage protective effects by downregulating the expression of SASP factors, mainly through deactivating NF-κB and PI3K/AKT signaling pathways (Wang et al., 2019). Subsequent studies reveal that it can halt OA progression by regulating Wnt/β-catenin and MAPK signaling pathways (Liu et al., 2020).

Vitexin, the active component isolated from hawthorn leaves, can deactivate NF-κB signaling pathway caused by endoplasmic reticulum stress (ERS) and downregulate the expression of SASP factors including IL-6, TNF-α, and MMP-3, which inhibits cartilage degradation and prevents OA progression in rats (Xie et al., 2018). In addition, vitexin alleviates IL-1β-mediated inflammatory response in chondrocytes derived from OA patients, significantly lowers the expression levels of IL-6, TNF-α, MMP-1, MMP-3, and MMP-13, and effectively protects chondrocytes (Yang et al., 2019).

Ruxolitinib, an FDA-approved JAK1/2 inhibitor, can ameliorate the senescence-related systemic inflammation in aged mice by suppressing the production of SASP factors in the SnCs via JAK/STAT pathway (Xu et al., 2015). Ruxolitinib may also prevent the age-related bone loss by down-regulating the expression of inflammatory SASP factors, such as IL-6, IL-8, PAI-1 (Farr et al., 2017). In addition, it inhibits the senescence-associated cell cycle arrest and suppresses the progeria-like phenotype in progeria mice (Griveau et al., 2020).

Metformin, a commonly used drug for type II diabetes (T2DM), demonstrates senomorphic activity. It attenuates pain and cartilage degeneration, and delays cartilage senescence in OA mice by activating AMPK signaling and inhibiting the mTOR signaling pathway, which causes downregulate expression of MMP-3, MMP-13, and upregulated expression of Col-II (Li H. et al., 2020; Li J. et al., 2020; Feng et al., 2020). Simvastatin inhibits the expression of MMP-3 in human OA chondrocytes cultured *in vitro* by blocking 3-hydroxy-3-methyl glutaryl coenzyme A reductase (HMG-CoA) and interfering with the prenylation process (Lazzerini et al., 2004). *In vitro*, simvastatin inhibits the MMP-1 and MMP-13 induced by IL-1β in human articular chondrocytes and prevents the senescence of chondrocytes (Yudoh and Karasawa, 2010). Intra-articular administration of simvastatin significantly reduces the expressions of MMP-13 and IL-1β in chondrocytes of OA mice, inhibits articular cartilage degeneration, and delays OA progression. It also activates autophagy signaling by decreasing mTOR phosphorylation in chondrocytes (Tanaka et al., 2019).

#### Senomorphics That Neutralize Specific SASP Components

In addition to these senomorphic drugs targeting SASP regulatory pathways mentioned above, other types of senomorphics are able to neutralize specific SASP components or their receptors, such as IL-1β, IL-6, and TNF-α, thereby preventing chondrocyte senescence. In fact, some monoclonal antibodies (mAbs) have already been used in clinic, and their senomorphic potential is promising in aging-related OA.

Canakinumab, a human mAb that neutralizes IL-1β, can significantly downregulate the expression of MMP-1, MMP-3, and MMP-13 in human OA chondrocytes induced by TNF-α and IL-1β by blocking IL-1β interaction with its receptor, exerting potential chondroprotective effects (Cheleschi et al., 2015). In an exploratory clinical trial, canakinumab reduced the overall hip or knee arthroplasty rate in patients with a history of OA at baseline (Schieker et al., 2020). This study suggests that IL-1β inhibition may be used as OA treatment. More studies are needed before its clinical use in OA.

Tocilizumab, a humanized mAb to IL-6R, can block the binding of IL-6 and IL-6R, and it is currently used in clinic to treat some different autoimmune rheumatoid diseases. IL-6 increases the production of MMP-3, MMP-13, ADAMTS-4, and ADAMTS-5 in chondrocytes, causing degradation of cartilage matrix proteins. Blocking IL-6 causes inhibition of cellular senescence, and alleviates the aging-related inflammation in premature senescent mice (Squarzoni et al., 2021). In addition, tocilizumab ameliorates allyodynia of rat OA (Lin et al., 2017). Another mAb to IL-6R attenuates cartilage damage and reduces osteophyte formation in mouse OA (Latourte et al., 2017).

Both etanercept and infliximab are TNF-α inhibitors, and they decrease the production of some SASP factors in human OA chondrocytes stimulated by TNF-α *in vitro*, including IL-6, IL-8, CCL-2, MMP-3, MMP-13, and ADAMTS-4 (Žigon-Branc et al., 2017). Etanercept reduces the serum levels of MMP-3 in patients with hand OA (Kroon et al., 2020). Besides, etanercept alleviates bone marrow lesions in hand OA patients, especially active joint inflammation already present at baseline, but its effect on synovitis-related pain is not evident (Kloppenburg et al., 2018). Adalimumab, an mAb to TNF-α, reduces MMP-13 expression, inhibits the degradation of cartilage matrix, and preserves the structure of articular cartilage in rat OA (Ma et al., 2015). In clinical trials, it effectively reduces pain and joint swelling in patients with knee OA (Maksymowych et al., 2012; Wang, 2018). However, clinical trials on hand OA do not yield satisfactory results with regards to pain-relieving and attenuating bone marrow lesions (Chevalier et al., 2015; Aitken et al., 2018). Similar to tocilizumab, the results from clinical trials of etanercept and adalimmumab in the treatment of hand OA are somewhat disappointing, warranting more studies.

Senomorphics on OA and other age-related diseases have been summarized in Table 3.

**TABLE 3:** Senomorphics on anti-aging therapy.

Senomorphics	Targets	Preclinical and clinical research of OA and others
Rapamycin	mTOR	Inhibited articular cartilage destruction in OA rats Bao et al. (2020).
Trichostatin A	HDAC	Reduced cartilage matrix degradation in OA mice Cai et al. (2015).
Scutellariae	NF-κB; PI3K/AKT	Inhibited articular cartilage damage in OA mice Wang et al. (2019).
Vitexin	NF-κB	Delayed the progression of OA rats Xie et al. (2018).
		Alleviated IL-1β-mediated inflammatory response in chondrocytes derived from OA patients Yang et al. (2019).
Ruxolitinib	JAK1/2	Alleviated aging-related systemic inflammation in aged mice Xu et al. (2015).
		Prevented age-related bone loss Farr et al. (2017).
Metformin	AMPK; mTOR	Attenuated pain and cartilage degeneration, and delayed cartilage senescence in OA mice Li et al (2020a); Li et al. (2020b); Feng et al. (2020).
Simvastatin	HMG-CoA; mTOR	Inhibited human articular chondrocyte senescence *in vitro* Yudoh and Karasawa (2010).
		Inhibited articular cartilage degeneration and delayed OA progression in mice Tanaka et al. (2019).
Canakinumab	IL-1β	Decreased MMPs expression in human OA chondrocytes Cheleschi et al., 2015).
		Reduced the overall hip or knee arthroplasty rate in patients with a history of OA at baseline Schieker et al. (2020).
Tocilizumab	IL-6 receptor	Reduced age-related inflammatory responses in premature aging mice Squarzoni et al. (2021).
		Attenuated central sensitization and pain in OA rats Lin et al. (2017).
		Failed in relieving pain in hand OA patient Richette et al. (2020).
Etanercept	TNF-α	Decreased some SASP expression in human OA chondrocytes *in vitro* Žigon-Branc et al. (2017).
		Reduced serum levels of MMP-3 in hand OA patients Kroon et al. (2020).
		Alleviated bone marrow lesions in hand OA patients, but failed in relieving synovitis and pain Kloppenburg et al. (2018).
Adalimmumab	TNF-α	Inhibited the degradation of cartilage matrix in OA rats Ma et al. (2015).
		Reduced pain and joint swelling in knee OA patients Maksymowych et al. (2012); Wang (2018).
		Failed in alleviating pain and bone marrow lesions in hand OA patients Chevalier et al. (2015); Aitken et al. (2018).

## Discussion

Our review summarizes the recent progresses in the biology related to the senescence of articular chondrocytes and novel anti-cellular senescence strategies for OA. Accumulating data suggest that OA is not a simple degenerative disease, instead, multifaceted factors are involved in its pathogenesis, including low-grade joint inflammation, and the senescence and autophagy of articular chondrocytes and synoviocytes. We focused on cellular senescence as this process plays a pivotal role in the development and progression of OA. Senescent chondrocytes secrete a variety of SASP components, including inflammatory cytokines, proteases, chemokines, etc., which may lead to destruction of joint integrity and aggravation of OA. There is an accumulation of SnCs in the articular cartilage from OA patients, and consistently, injection of the senescent chondrocytes into mouse knee joints induces OA-like changes. Removal of these SnCs may alleviate OA symptoms, lower the levels of the SASP factors, relieve joint pain, and prevent cartilage destruction. Collectively, these studies suggest a critical role of cell senescence and SASP factors in the pathogenesis of OA, and anti-cellular senescence strategies hold great promise for the treatment of OA. In fact, some anti-cellular senescence drugs have been trialed in OA, and the results show that some of them are inspiring, whereas others have been halted during efficacy and safety issues. Modifications of these agents or re-design of the clinical trials are needed to get reproducible data supporting their potential use in OA. Several critical questions need to be answered. Firstly, as aging plays an important role in many age-related diseases, it is reasonable to postulate that different types of SnCs may have a leading role in different diseases, and that senescent chondrocytes may be the major cells responsible for OA formation. Indeed, previous studies have shown that senescent chondrocytes can induce or exacerbate OA. Secondly, different types of SnCs may have different survival pathways, and senolytics drugs targeting specific molecules in specific survival pathways may be more effective in the elimination of senescent cells. As SA-β-Gal is a marker of senescent cells and can be used as prodrug to accurately kill SnCs. More Gal-like drug delivery systems are expected to be developed in order to obtain the systems with high specificity and less off-target effects. In addition, senomorphics should be optimized based on SASP secreted by senescent chondrocytes and the pathway regulating the production of SASP factors such as IL-1β, IL-6, MMP-3, and ADAMTS-5. Thirdly, does the combined therapy with STACs, senolytics, and senomorphics have better efficacy than a single drug in OA treatment? Improved understanding of the pharmacokinetics and interactions of these drugs may help answer this question. We expect that the combined therapy may have a higher efficacy in OA treatment, but the incidence of adverse effects might also increase. Finally, which mode of treatments will maximize the treatment outcome, intermittent or continuous? Since it takes several weeks for SnCs to re-accumulate, intermittent rather than long-term administration could be adopted to reduce off-target damage to normal cells (Farr et al., 2017). Meanwhile, intermittent administration may reduce the risk of adverse reactions and the possibility of drug resistance (Khosla et al., 2020). However, the modes of administration of senomorphics still remain a matter of debate. Animal experiment indicates that intermittent administration is sufficient (Laberge et al., 2015). However, long-term administration may cause off-target effects, because of the multiple mechanisms involved in SASP inhibitors (Khosla et al., 2020). Controversy still remains regarding the modes of administration of these SASP inhibitors, whereas it is well accepted that senolytics should be given intermittently. Further animal studies and clinical trials are needed to determine the efficacy and safety profiles of intermittent vs continuous administration, and local vs systemic administration. In summary, senolytics able to kill SnCs may have some advantages over senomorphics as the latter cannot induce apoptosis of SnCs. Senolytics can be given intermittently to avoid the potential harmful effects after long-term administration.

Whether in preclinical research or ongoing clinical trials, these anti-cellular senescence strategies show great potential for the treatment of OA. More clinical trials are needed to determine the feasibility and reliability of anti-cellular senescence drugs. Great endeavors should be made to optimize these drugs before clinical use in OA.

## Conclusion

OA is the most common type of chronic debilitating disease, and no DMOADs are currently available. Preventing OA formation and progression and preserving joint structure remain an unmet medical need. Emerging data suggest that aging and cell senescence are of critical importance in OA pathogenesis. Intensive studies have been conducted to discover and develop the anti-cellular senescence drugs targeting senescent chondrocytes. They have also been continuously discovered and developed, and have shown considerable potential in preclinical studies to delay senescence, reduce senescence-related phenotypes, and improve senescence-related symptoms. And some anti-cellular senescence drugs for the treatment of OA and other diseases have now entered clinical trials. This emerging anti-cellular senescence strategy holds considerable future clinical promise and may become a revolutionary OA treatment modality.

## Abbreviations

OA, osteoarthritis; DMOADs, disease modifying anti-osteoarthritis drugs; STACs, sirtuin-activating compounds; NSAIDs, non-steroidal anti-inflammatory drugs; SnCs, Senescent cells; SCAPs, senescent cell anti-apoptotic pathways; SA-β-Gal, senescence-associated-β-galactosidase; SAHFs, senescence-associated heterochromatic foci; MMP, matrix metalloproteinase; SASP, senescence-associated secretory phenotype; NF-κB, nuclear factor-κB; ECM, extracellular matrix; ADAMTS, a disintegrin and metalloproteinase with thrombospondin motifs; MAPK, mitogen-activated protein kinase; PPARα, peroxisome associator-activated receptor-α; SIRT, sirtuin; Col-II, type II collagen; AMPK, adenosine 5 ′-monophosphate (AMP) -activated protein kinase; HSP90, heat shock protein 90; JAK/STAT, the janus kinase/signal transducer and activator of tranions; mTOR, mammalian target of rapamycin; PI3K, phosphatidylinositol 3-kinase; HDACi, Histone deacetylase inhibitor
